# Hospital-Associated Disability Associated With Delirium Among Older Adults

**DOI:** 10.1093/geroni/igab046.2232

**Published:** 2021-12-17

**Authors:** Richard Kennedy, Hyun Freeman, Roy Martin, Caroline Whittington, John Osborne, Alayne Markland, Thomas Buford, Cynthia Brown

**Affiliations:** 1 University of Alabama at Birmingham, Birmingham, Alabama, United States; 2 University of Alabama at Birmingham, University of Alabama at Birmingham, Alabama, United States; 3 Louisiana State University Health Sciences Center - New Orleans, New Orleans, Louisiana, United States

## Abstract

Hospital-associated disability (HAD), defined as a loss of activities of daily living (ADLs) occurring during hospitalization, is a common complication among older adults. Delirium is also a common complication during hospitalization and is associated with multiple long-term sequelae. We sought to determine the effect of delirium and known covariates on the risk of incident HAD in hospitalized older adults. We examined electronic health record (EHR) data for 35,201 older adults ≥ 65 years of age admitted to the general inpatient (non-ICU) units of UAB Hospital from January 1, 2015 to December 31, 2019. Delirium was defined as a score ≥ 2 on the Nursing Delirium Screening Scale (NuDESC) during hospital admission, and HAD defined as a decline on the Katz ADL scale from hospital admission to discharge. Generalized linear mixed models were used to examine the association between delirium and HAD, adjusting for covariates and repeated observations for individuals with multiple admissions. We found that 21.2% of older adults developed HAD during their hospitalization and experienced higher delirium rates as compared to those not developing HAD (25.2% vs. 16.3%). Presence of delirium, medical comorbidity score, baseline cognitive status, and baseline ADL function were associated (all p <0.001) with incident HAD. Mediation analyses also showed that 8% of the effect of comorbidity on incident HAD was due to delirium (p < 0.001). Reducing rates of delirium can be one component of a comprehensive approach to reduce rates of HAD in older adults.

